# Railway Intrusion Risk Quantification with Track Semantic Segmentation and Spatiotemporal Features

**DOI:** 10.3390/s25175266

**Published:** 2025-08-24

**Authors:** Shanping Ning, Feng Ding, Bangbang Chen, Yuanfang Huang

**Affiliations:** 1 School of Mechatronic Engineering, Xi’an Technological University, Xi’an 710016, China; ningshanping@163.com (S.N.); chenbangbang@st.xatu.edu.cn (B.C.); 2 Railway Transportation Institute, Guangdong Communication Polytechnic, Guangzhou 510650, China; hyfang003@163.com

**Keywords:** foreign object intrusion, risk quantification assessment, semantic segmentation, spatiotemporal feature fusion, lightweight network, graded early warning

## Abstract

Foreign object intrusion in railway perimeter areas poses significant risks to train operation safety. To address the limitation of current visual detection technologies that overly focus on target identification while lacking quantitative risk assessment, this paper proposes a railway intrusion risk quantification method integrating track semantic segmentation and spatiotemporal features. An improved BiSeNetV2 network is employed to accurately extract track regions, while physical-constrained risk zones are constructed based on railway structure gauge standards. The lateral spatial distance of intruding objects is precisely calculated using track gauge prior knowledge. A lightweight detection architecture is designed, adopting ShuffleNetV2 as the backbone to reduce computational complexity, with an incorporated Dilated Transformer module to enhance global context awareness and sparse feature extraction, significantly improving detection accuracy for small-scale objects. The comprehensive risk assessment formula integrates object category weights, lateral risk coefficients in intrusion zones, longitudinal distance decay factors, and dynamic velocity compensation. Experimental results demonstrate that the proposed method achieves 84.9% mean average precision (mAP) on our proprietary dataset, outperforming baseline models by 3.3%. By combining lateral distance detection with multidimensional risk indicators, the method enables quantitative intrusion risk assessment and graded early warning, providing data-driven decision support for active train protection systems and substantially enhancing intelligent safety protection capabilities.

## 1. Introduction

Railways, as critical national transportation infrastructure, play a strategic supporting role in passenger mobility, freight transportation, and regional economic development [1]. However, the complex and variable operational environment makes foreign object intrusion a significant threat to railway safety, particularly the incursion of pedestrians, falling rocks, wildlife, and other objects within the railway clearance zone [2]. Such safety incidents may trigger catastrophic consequences, including train derailments, equipment damage, and other major accidents, resulting in severe casualties and substantial economic losses [3]. Therefore, assessing intrusion risks in railway perimeter areas is crucial for ensuring operational safety.

**Figure 1:**
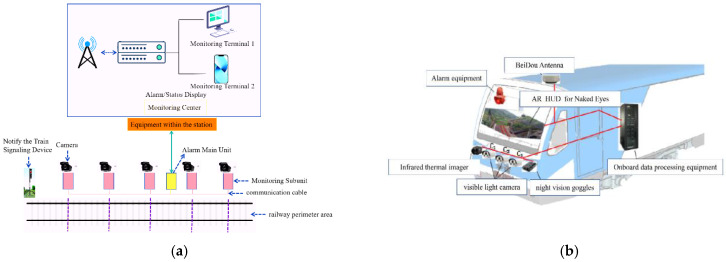
Non-contact vision-based detection technology. (**a**) Wayside installation schematic diagram. (**b**) Onboard equipment installation schematic diagram.

As summarized in [4], current intrusion detection technologies for track clearance zones can be classified into contact-based and non-contact systems. Contact-based detection primarily relies on physical sensors such as vibration optical fibers [5] and pulse electronic fences [6], which perceive intrusions through physical interactions. Non-contact detection employs vision-based recognition [7], radar sensing [8], and other non-intrusive sensors, achieving precise target identification and localization through multi-dimensional environmental perception and intelligent algorithm fusion. As illustrated in Figure 1, vision-based detection in railway intrusion monitoring mainly adopts two approaches: wayside-installed and onboard systems. The former constructs a comprehensive detection network via densely deployed visual sensors but suffers from high infrastructure costs and complex maintenance. The latter utilizes onboard intelligent camera systems to enable dynamic environmental perception during train operation, offering advantages in real-time performance, wide coverage, and cost efficiency, making it a more promising solution for railway intrusion detection [9].

In the development of intelligent railway safety technologies, while deep learning-based visual detection systems have made breakthroughs in foreign object identification—as demonstrated by Chen et al.’s [10] efficient two-stage framework for railway image object detection with significant efficiency improvements and Meng et al.’s [11] high-performance intrusion detection method addressing the limitations of traditional approaches in complex railway environments, including high false-alarm rates, missed detections, and poor real-time performance—current research still faces notable limitations. As noted in [12], existing studies predominantly focus on optimizing detection model accuracy and real-time performance while lacking systematic research on quantitative safety risk assessment of intrusion behaviors. This highlights the urgent need to establish a physically constrained risk evaluation framework, which presents two key technical challenges: (1) integrating high-precision semantic segmentation networks with railway clearance standards to develop a physically constrained track boundary model for spatial relationship analysis between intruding objects and safety limits and (2) formulating a comprehensive risk assessment model that incorporates multidimensional features such as object category, intrusion zone, and lateral/vertical distances to establish a risk entropy calculation model [13]. Such a risk-quantified intelligent decision-making mechanism enables the advancement from simple object detection to quantitative risk assessment, thereby enhancing the proactive defense capabilities and operational efficiency of railway security systems [14]. To address these challenges, this paper innovatively proposes a railway intrusion risk quantification method that integrates track semantic segmentation and spatiotemporal features, featuring an improved BiSeNetV2 network for precise track detection, a physically constrained risk zone model, a lightweight ST-YOLO architecture for efficient intrusion detection, and a five-level risk assessment system incorporating multidimensional factors, significantly improving railway safety protection capabilities. The main contributions of this study can be summarized as follows:

- A physically constrained track boundary model was developed by integrating railway clearance standards with an enhanced semantic segmentation network. This model incorporates track prior knowledge and enables spatial distance calculation of foreign objects through pixel transformation.
- An ST-YOLO based heterogeneous feature fusion mechanism was proposed, where the original YOLOv8s backbone network was replaced with an improved lightweight ShuffleNetV2 architecture. A DilateFormer module (Dilated Transformer) was incorporated at the bottom of the backbone network to significantly enhance feature extraction capability.
- A dynamic risk assessment matrix was constructed by integrating three-dimensional feature vectors: intruding object category, intrusion zone weighting coefficient, and spatiotemporal distance threat value. This enables comprehensive quantitative risk evaluation of foreign object intrusions.

The remainder of this paper is organized as follows: Section 2 presents the experimental methodology, including track detection, foreign object identification, and risk assessment level modeling. Section 3 demonstrates the experimental results and validates the effectiveness of the proposed approach through comparative analysis. Finally, Section 4 summarizes the key findings and suggests potential directions for future research.

## 2. Foreign Object Detection and Risk Assessment

**Figure 2:**
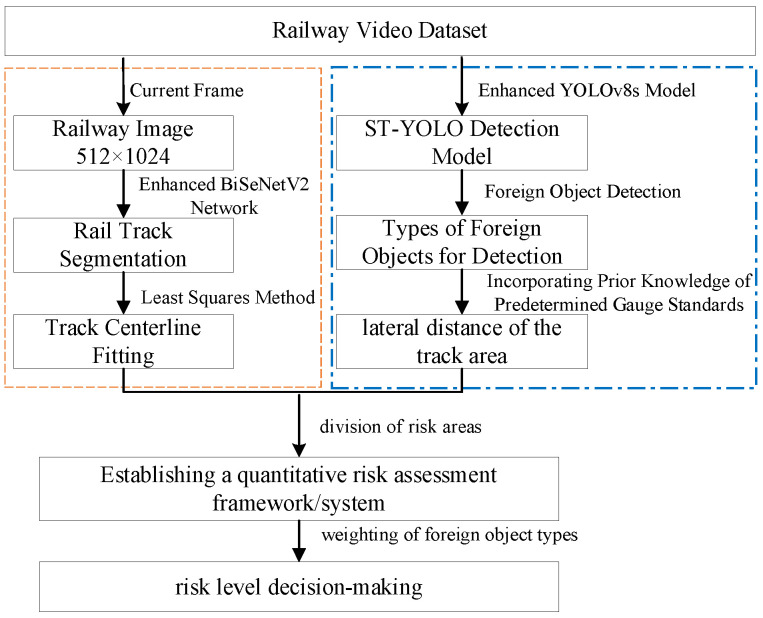
Framework diagram of this paper.

This section presents our integrated methodology for target detection, intrusion determination, and risk assessment using onboard train image data. The framework first employs the BiSeNetV2 model for precise track identification, subsequently expanding the detected boundaries according to railway structural clearance safety standards to establish the critical clearance zone. Building upon this foundation, we implement an enhanced YOLO-based algorithm for robust object detection. The system’s innovation lies in its risk assessment module, which synthesizes real-time spatial configurations and object hazard levels to generate comprehensive risk classifications for intrusion events. The flowchart is shown in Figure 2. The resulting evaluations provide unified, rapid alerts to train operators, control centers, and relevant authorities, enabling immediate emergency response measures when necessary.

### 2.1. Track Detection Based on BiSeNetV2 Semantic Segmentation Network

In general, image-based track detection methods can be classified into traditional algorithms and deep learning approaches [15]. Conventional methods for railway track detection typically utilize the Hough transform for rail identification, which effectively detects linear features through parameter space conversion [16]. However, when the track curvature radius decreases or continuous curved segments are present, discontinuous distribution of edge pixels occurs, resulting in weakened gradient direction information and consequently causing edge fragmentation and trajectory discontinuity in detection results. Deep learning methods address these limitations by constructing deep convolutional neural network models that leverage their adaptive feature extraction capabilities and superior complex curve fitting performance, thereby significantly improving detection accuracy and robustness for curved tracks. Based on these advantages, this study employs the lightweight BiSeNetV2 semantic segmentation network for track region extraction [17].

**Figure 3:**
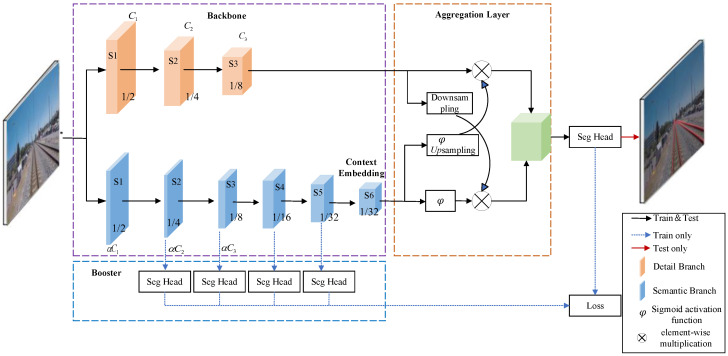
Architecture of the BiSeNetV2 Network.

BiSeNetV2 employs a dual-branch backbone architecture comprising a Detail Branch and a Semantic Branch [18]. The Detail Branch is designed with wide channels and shallow layers to capture low-level spatial details, while the Semantic Branch features narrow channels and deep layers for extracting high-level semantic information. The features from both branches are fused through a Bilateral Guided Aggregation Layer (GA Layer), with final segmentation predictions generated by the Segmentation Head (Seg Head). The Seg Head consists of a 3 × 3 convolutional layer followed by a 1 × 1 convolutional layer [19]. The overall architecture is illustrated in Figure 3.

In the Detail Branch, which focuses exclusively on low-level details, three groups of small-stride convolutional blocks are implemented. Each block contains two standard convolutional layers (Conv + BN + ReLU) with strides of 2 and 1, respectively, halving the feature map resolution while doubling the channel dimensionality. The Semantic Branch initiates with two distinct downsampling operations (Stem blocks) to rapidly reduce spatial dimensions and expand receptive fields. Subsequently, Gather-and-Expansion (GE) layers perform feature extraction and aggregation, compressing high-dimensional feature responses into lower-dimensional spaces. Finally, a Context Embedding (CE) block incorporating global average pooling and residual connections captures global contextual semantics. The detailed network configurations for both branches are specified in Table 1.

**Figure 4:**
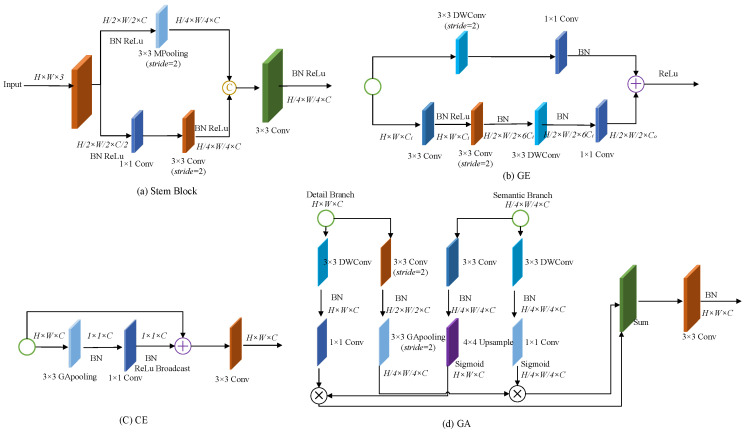
Detailed architecture of BiSeNetV2 submodules.

In Table 1, each stage *S* comprises one or more operations (e.g., Conv2d, Stem, GE, CE). Each operation employs a kernel of size *k*, a stride of *s*, and produces *c* output channels, repeated *r* times. An expansion factor *e* is utilized to expand the channel count of the operation (with a channel ratio λ = 1/4). In the corresponding stage of the detail branch, green highlights indicate fewer channels in the semantic branch. Note: Conv2d denotes a convolutional layer followed by a batch normalization layer and a ReLU activation function. GE represents a Gather-Expand layer. CE stands for Context Embedding block. In the fusion stage, BiSeNetV2 incorporates a Bilateral Guided Aggregation (GA) Layer to merge outputs from the Detail Branch and Semantic Branch. In contrast to simplistic fusion approaches, the GA Layer enables more efficient information exchange and complementary feature integration. The structural details of the Stem blocks, Gather-and-Expansion (GE) modules, Context Embedding (CE) blocks, and GA Layer are depicted in Figure 4.

**Figure 5:**
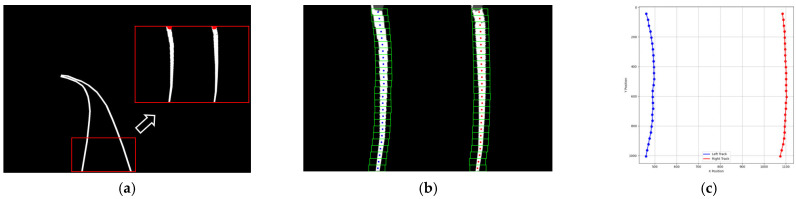
Fitting results of left and right rail tracks. (**a**) Track segmentation result. (**b**) Sliding windows with track points. (**c**) Track points distribution.

Additionally, BiSeNetV2 incorporates an auxiliary loss function during training to guide parameter updates in intermediate layers of the Semantic Branch, thereby enhancing its feature representation capabilities. Figure 5a demonstrates the track segmentation results of BiSeNetV2 in railway scenarios. A sliding window approach is employed to detect rail points within the red highlighted region of Figure 5a. As shown by the green rectangular windows in Figure 5b, the search initiates from two starting points and proceeds until all image rows are processed. All white points captured within these windows, illustrated in Figure 5c, constitute the candidate points for subsequent rail line fitting.

The candidate fitting points were processed using a cubic polynomial least squares fitting method to generate the rail tracks, with the computational formula expressed as follows:

(1) $$f\left(x\right)=a_{0}+a_{1}x+a_{2}x^{2}+a_{3}x^{3}$$

The four positional parameter coefficients in Equation (1) are determined by minimizing the sum of squared errors to achieve optimal functional fitting to the data. Given the known data points $\left(x_{i},y_{i}\right)$, where *i* = 1, 2, …, *n*, the sum of squared errors *Q* for the fitted curve is expressed as follows:

(2) $$Q=\sum_{i=1}^{n}(a_{0}+a_{1}x_{i}+a_{2}x_{i}^{2}+a_{3}x_{i}^{3}-y_{i}{)}^{2}$$

To minimize the sum of squared errors, partial derivatives of both sides of the equation with respect to coefficients $a_{0}$, $a_{1}$, $a_{2}$, and $a_{3}$ are calculated. The first-order derivatives for these four variables should equal zero, yielding the following system of equations:

(3) $$\left\{\begin{array}{l} \frac{\partial Q}{\partial a_{0}}=\sum_{i=1}^{n}2\left[f(x_{i})-y_{i}\right]=0\Rightarrow \sum_{i=1}^{n}f(x_{i})=\sum_{i}^{n}y_{i}\\ \frac{\partial Q}{\partial a_{1}}=\sum_{i=1}^{n}2x_{i}\left[f(x_{i})-y_{i}\right]=0\Rightarrow \sum_{i=1}^{n}x_{i}f(x_{i})=\sum_{i=1}^{n}x_{i}y_{i}\\ \frac{\partial Q}{\partial a_{2}}=\sum_{i=1}^{n}2x_{i}^{2}\left[f(x_{i})-y_{i}\right]=0\Rightarrow \sum_{i=1}^{n}x_{i}^{2}f(x_{i})=\sum_{i=1}^{n}x_{i}^{2}y_{i}\\ \frac{\partial Q}{\partial a_{3}}=\sum_{i=1}^{n}2x_{i}^{3}\left[f(x_{i})-y_{i}\right]=0\Rightarrow \sum_{i=1}^{n}x_{i}^{3}f(x_{i})=\sum_{i=1}^{n}x_{i}^{3}y_{i} \end{array}\right.$$

Equation (3) can be transformed into matrix form as follows:

(4) $$\left[\begin{array}{cccc} n & \sum_{i=1}^{n}x_{i} & \sum_{i=1}^{n}x_{i}^{2} & \sum_{i=1}^{n}x_{i}^{3}\\ \sum_{i=1}^{n}x_{i} & \sum_{i=1}^{n}x_{i}^{2} & \sum_{i=1}^{n}x_{i}^{3} & \sum_{i=1}^{n}x_{i}^{4}\\ \sum_{i=1}^{n}x_{i}^{2} & \sum_{i=1}^{n}x_{i}^{3} & \sum_{i=1}^{n}x_{i}^{4} & \sum_{i=1}^{n}x_{i}^{5}\\ \sum_{i=1}^{n}x_{i}^{3} & \sum_{i=1}^{n}x_{i}^{4} & \sum_{i=1}^{n}x_{i}^{5} & \sum_{i=1}^{n}x_{i}^{6} \end{array}\right]\left[\begin{array}{c} a_{0}\\ a_{1}\\ a_{2}\\ a_{3} \end{array}\right]=\left[\begin{array}{c} \sum_{i=1}^{n}y_{i}\\ \sum_{i=1}^{n}x_{i}y_{i}\\ \sum_{i=1}^{n}x_{i}^{2}y_{i}\\ \sum_{i=1}^{n}x_{i}^{3}y_{i} \end{array}\right]$$

The coefficients $a_{0}$, $a_{1}$, $a_{2}$, and $a_{3}$ in Equation (4) are subsequently solved using Cramer’s rule. These determined coefficients are then substituted into Equation (1) to obtain the cubic polynomial expression derived from the least squares method, thereby achieving optimal fitting of the rail tracks [20].

### 2.2. ST-YOLO Foreign Object Detection Model

#### 2.2.1. ShuffleNetV2 Network and Its Enhancements

**Figure 6:**
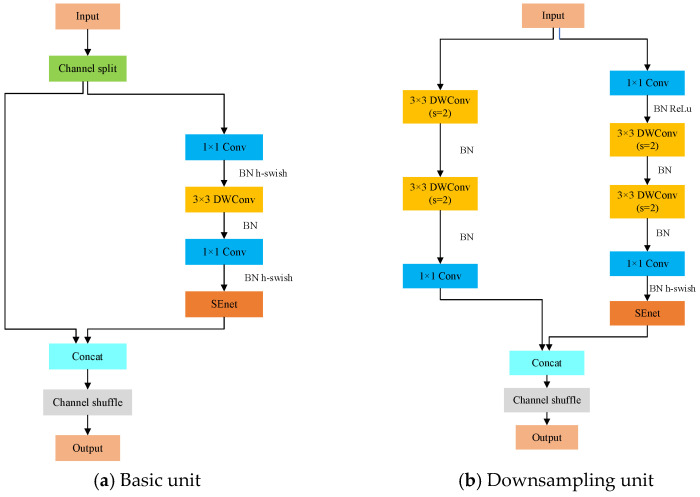
Architecture of the enhanced ShuffleNetV2 network.

ShuffleNet represents a lightweight network architecture whose core innovation lies in employing grouped pointwise convolutions to dramatically reduce parameters, while introducing channel shuffle operations to enhance cross-group information exchange and fusion. This design achieves significant computational efficiency gains while effectively maintaining model accuracy [21]. Compared to networks like MobileNetV3 [22], Faster R-CNN [23], and AlexNet [24], ShuffleNet’s architecture demonstrates superior specialization for efficiency optimization. Building upon ShuffleNetV1, ShuffleNetV2 successfully addresses four critical technical limitations, delivering further performance enhancements. Consequently, our study adopts ShuffleNetV2, which synergistically integrates grouped pointwise convolutions, channel shuffle strategies, and depthwise separable convolutions to achieve higher computational efficiency without compromising accuracy. The ShuffleNetV2 architecture primarily consists of basic units and downsampling units, with our enhanced version’s structure detailed in Figure 6.

**Figure 7:**
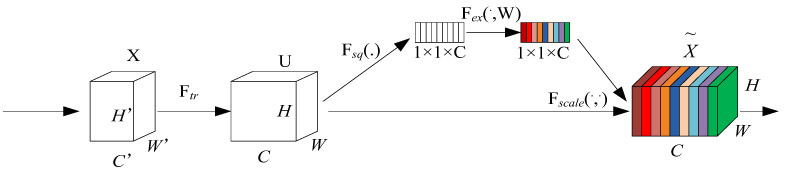
Architecture of the Squeeze-and-Excitation (SE) attention module.

The core innovation of the Squeeze-and-Excitation Network (SEnet) attention mechanism lies in its adaptive learning capability to automatically evaluate and quantify the importance of each feature channel. This mechanism dynamically recalibrates original features using learned channel-wise importance weights, amplifying responses from critical feature channels while suppressing less relevant ones, thereby enhancing task-specific feature representations. These operations are collaboratively achieved through its two fundamental modules: the Squeeze module and the Excitation module [25]. The detailed architecture of the SEnet attention mechanism is illustrated in Figure 7.

To enhance model accuracy while maintaining minimal computational overhead, we integrate the Squeeze-and-Excitation (SE) attention module after the 1 × 1 convolutional kernels in the backbone network (as illustrated in Figure 5). This strategic incorporation of the SE mechanism within ShuffleNetV2 enables more precise detection and localization of foreign objects in images by adaptively emphasizing relevant spatial-channel features.

#### 2.2.2. Foreign Object Detection Model

**Figure 8:**
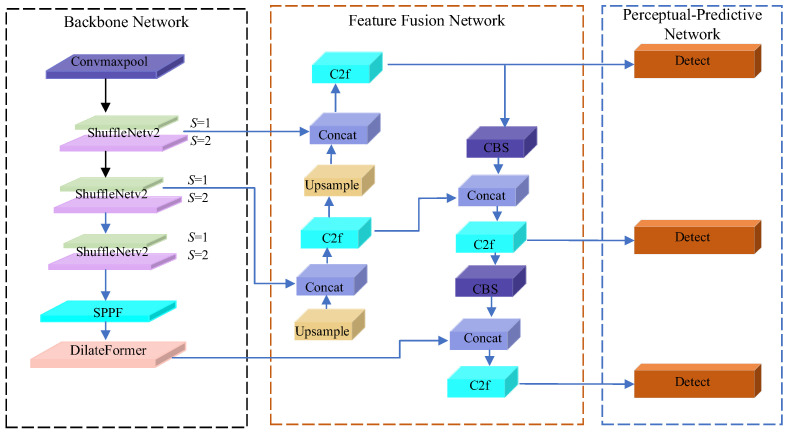
Architecture of the ST-YOLO network.

To address the excessive parameter size and computational complexity of the DarkNet-53 backbone network in YOLOv8 [26], this section proposes a lightweight ST-YOLO detection model for rapid and accurate identification of foreign objects on railway tracks. The ST-YOLO model primarily replaces YOLOv8s’ original backbone with a lightweight ShuffleNetV2 network. As feature maps undergo multiple downsampling operations in the backbone network, their resolution decreases significantly. To prevent loss of detailed information, an efficient Dilated Transformer (DilateFormer) module is incorporated at the deepest layer of the backbone network. This module endows the network with sliding sparse sampling characteristics, reducing fine-grained information loss while maintaining higher feature resolution. Additionally, it better utilizes multi-scale feature information extracted from preceding layers, thereby enhancing the network’s feature extraction capability. These innovations enable the model to maintain high accuracy while substantially optimizing both parameter quantity and computational complexity. The architecture of ST-YOLO is illustrated in Figure 8.

**Figure 9:**
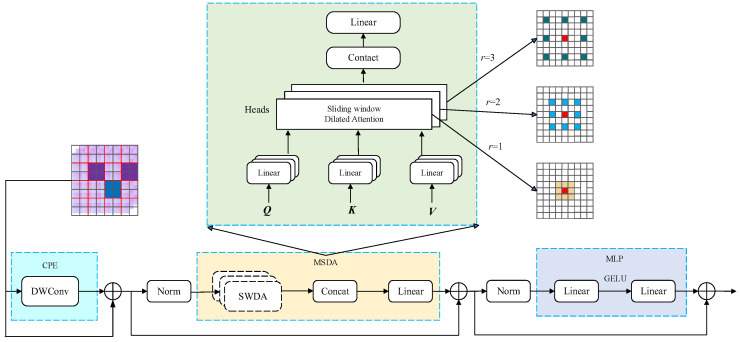
Architecture of the DilateFormer network.

Transformer models have demonstrated remarkable potential in computer vision tasks due to their powerful global context modeling capability, adaptability to variable-sized inputs, and simultaneous learning of both global and local features. However, conventional Transformers suffer from high computational complexity and information redundancy. To address these limitations, this study introduces an efficient Dilated Transformer (DilateFormer) structure at the terminal stage of the backbone network. The proposed architecture consists of Multi-Scale Dilated Attention (MSDA) modules that employ a hierarchical dilation mechanism to effectively reduce computational overhead while minimizing redundant information, without compromising multi-scale feature extraction capability [27]. The detailed architecture of DilateFormer is presented in Figure 9.

The MSDA module employs a sliding-window attention mechanism where, for each query patch, it selectively samples the most representative key and value patches within a centered sliding window, rather than computing global attention [28]. This design significantly reduces computational complexity while effectively capturing both local and cross-window dependencies, as mathematically represented below:

(5) X=SWDA(Q,K,V,r)

In Equation (5), *Q*, *K*, and *V* represent the query, key, and value matrices, respectively, while *r* denotes the coefficient controlling sparsity degree. For a query at unknown position (i,j), the SWDA mechanism selectively computes self-attention using only keys and values within a sliding window of size w×w centered at (i,j). Mathematically, the output *X* of SWDA at position (i,j) can be expressed as follows:

(6) $$x_{ij}=Attention(q_{ij},K_{r},V_{r})=Soft\max(\frac{q_{ij}K_{r}^{T}}{\sqrt{d_{k}}})V_{r}$$

In Equation (6), 1≤i≤W, 1≤j≤H, $q_{ij}$ denotes the query vector at position (i,j) of the feature map, while $K_{r}$ and $V_{r}$ represent the sampled values from *K* and *V*, respectively. These parameters further enable the derivation of key and value expressions at position $\left(i^{\prime },j^{\prime }\right)$ for self-attention computation:

(7) $$\left\{(i^{\prime }\right.,\;j^{\prime })\left|i^{\prime }\right.=i+pr,j^{\prime }=j+\left.qr\right\}$$

In Equation (7), $-\frac{w}{2}\leq p$, $q\leq \frac{w}{2}$, the SWDA mechanism achieves both locality and sparsity by selectively choosing only relevant keys and values around each query, thereby significantly reducing computational overhead.

To capture multi-scale features, the MSDA module splits the input feature map along the channel dimension into multiple heads, where each head applies distinct dilation coefficients to sample patches surrounding the queries. The algorithm then aggregates feature representations from all heads through concatenation, followed by final transformation via a linear projection layer. This process can be mathematically represented as follows:

(8) $$h_{i}=SWDA(Q_{i},K_{i},V_{i},r_{i});1\leq i\leq n$$

In Equation (8), $r_{i}$ denotes the *i*-th dilation coefficient, $Q_{i}$, $K_{i}$, $V_{i}$ represents the feature map of the *i*-th head, and *n* is an integer specifying the number of heads in the multi-head self-attention mechanism when dividing the feature map’s channels. MSDA (Multi-Scale Dilated Attention) achieves multi-scale semantic perception through a hierarchical dilated mechanism, and its effectiveness relies on two fundamental assumptions: First is the locality assumption, which posits that spatially adjacent pixels exhibit strong correlations, enabling sparse sampling within sliding windows to retain over 90% of key features. Second is the scale-decoupling assumption, which states that features at different semantic scales can be processed separately, with each head focusing on a specific scale after channel splitting. Compared to traditional Transformers, MSDA can effectively extract semantic information across different scales while reducing fine-grained information loss and redundancy in global attention computations.

### 2.3. Spatiotemporal Distance-Based Risk Quantification for Foreign Object Intrusion

#### 2.3.1. Lateral Distance Calculation Between Obstacles and Track Area

**Figure 10:**
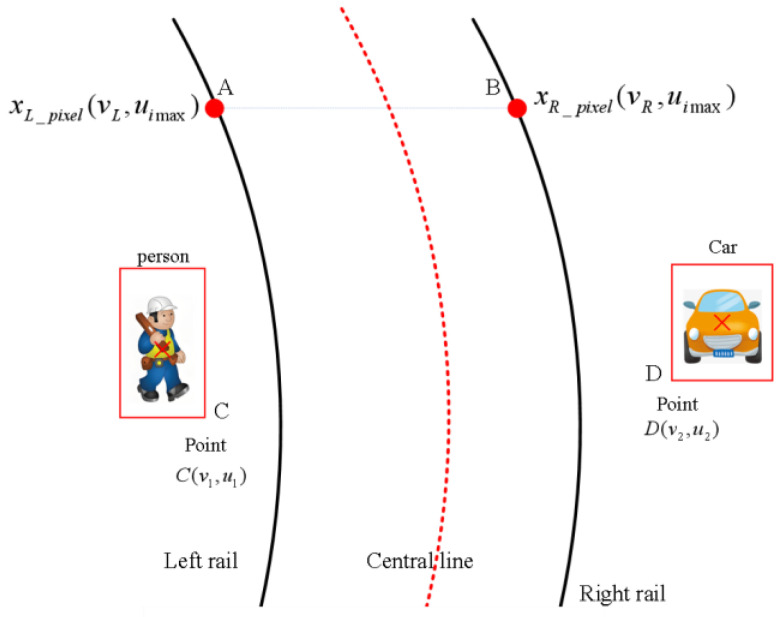
Coordinate correspondence between obstacle contour points and track contour points.

In Chinese railway operations, by incorporating prior knowledge of standard gauge specifications [29], the track gauge is maintained at a fixed standard value *d_m_ =* 1435 mm, as illustrated in Figure 10.

In the figure, the two track endpoints sharing the same vertical coordinate at points *A* and *B* are designated as $x_{L\_pixel}(v_{L},u_{i\max})$ and $x_{R\_pixel}(v_{R},u_{i\max})$, respectively. The Euclidean distance between endpoints *A* and *B* in the image coordinate system is calculated as follows:

(9) $$d_{p}=\left|x_{R\_pixel}-x_{L\_pixel}\right|=\left|v_{R}-v_{L}\right|$$

In the equation, the unit of *d_p_* is pixels. Consequently, the conversion ratio (calibration coefficient) between pixel and physical distance is expressed as

(10) $$k=\frac{d_{m}}{d_{p}}=\frac{1435}{\left|v_{R}-v_{L}\right|}\;\mathrm{mm}/\mathrm{pixel}$$

Taking the track symmetry center in the image as the origin *O* (in pixel coordinates), the horizontal coordinate of origin *O* can be calculated as

(11) $$x_{o\_pixel}=\frac{\left|x_{R\_pixel}+x_{L\_pixel}\right|}{2}=\frac{\left|v_{R}+v_{L}\right|}{2}$$

Assuming the detected foreign object point *C* with pixel coordinates $C(v_{1},u_{1})$, its pixel offset relative to the track symmetry center is calculated as

(12) $$\Delta x_{pixel}=v_{1}-x_{o\_pixel}$$

The pixel offset is converted to physical lateral distance using calibration coefficient *k* as follows:

(13) $$\Delta x_{actual}=k\times \Delta x_{pixel}$$

Thus, the distances from the foreign object to both left and right tracks can be expressed as

(14) $$\left\{\begin{array}{c} d_{L}=\left|\Delta x_{actual}+\frac{1435}{2}\right|\\ d_{R}=\left|\Delta x_{actual}-\frac{1435}{2}\right| \end{array}\right.$$

#### 2.3.2. Construction of Risk Quantification Assessment System

The proposed intrusion detection and track feature extraction methods enable precise localization of foreign objects relative to track lanes. By integrating these positional measurements with the standard rail gauge (1435 mm) and structural clearance limits (4880 mm) defined in Chinese National Standard GB-163.2020 [30], we establish the risk classification zones, as systematically detailed in Table 2.

Based on Table 1, we develop a dynamic risk assessment model in which the lateral position risk coefficient $x_{risk}$ is determined by the regional category, as expressed by

(15) $$x_{risk}=\left\{\begin{array}{c} 1-0.3(\frac{d_{x}}{717.5})0\leq d_{x}\leq 717.5\\ 0.5(1-\frac{\left(d_{x}-717.5\right)}{2440})717.5\leq d_{x}\leq 3157.5\\ 0d_{x}\geq 3157.5 \end{array}\right.$$

In the formula, $x_{risk}$ is the lateral position risk coefficient, and $d_{x}$ is the distance of the foreign object from the center of the track.

The longitudinal distance risk coefficient $y_{risk}$ adopts a piecewise function to amplify near-field risks [31], giving

(16) $$y_{risk}=\left\{\begin{array}{c} 1.5\times e^{-0.5d_{y}}d_{y}\leq 20m\\ e^{-0.2d_{y}}d_{y}\geq 20m \end{array}\right.$$

In the formula, $y_{risk}$ represents the longitudinal distance risk coefficient, and $d_{y}$ denotes the longitudinal distance of the foreign object from the camera.

Meanwhile, according to the literature [32], the primary foreign objects that intrude during train operation include vehicles, pedestrians, animals, and falling rocks. Based on the behavioral characteristics of different objects and in combination with the literature [33], the determined weights are listed in Table 3.

To conclude, the integrated risk assessment formula for intrusive objects may be formulated as

(17) $$Z_{risk}=x_{risk}\times y_{risk}\times W_{type}\times f_{speed}$$

In the given equation, $Z_{risk}$ corresponds to the integrated risk quantification value, $W_{type}$ represents the categorical weighting coefficient, and $f_{speed}$ stands for the dynamic velocity compensation factor, where $f_{speed}$ = 1 + *v*/10 applies to moving objects while $f_{speed}$ = 0 for stationary objects [34].

Based on the risk values calculated using formula 17, a risk level decision table has been constructed, as shown in Table 4. This table categorizes risks into five levels and establishes corresponding response strategies: when the risk value is ≥1.2, it is classified as extremely high risk (Level I), requiring emergency braking; when the risk value falls within the range of 0.8 to 1.2, it is considered high risk (Level II), corresponding to mandatory speed restriction measures; the range of 0.5 to 0.8 indicates a relatively high risk (Level III), necessitating the triggering of audible and visual alarms and preparation for braking; the interval of 0.2 to 0.5 represents general risk (Level IV), with warnings provided via HUD; and when the risk value is <0.2, it is deemed low risk (Level V), requiring only log recording. This decision table provides a quantifiable basis for risk management and control in railway operational safety through the quantification of risk thresholds and a graded response mechanism.

## 3. Experiments

The experimental hardware configuration comprised a 13th Gen Intel^®^ Core™ i7-13700 processor (USA) and an NVIDIA RTX A4000 graphics card (USA) with 16 GB GDDR6 memory. The software environment included Windows 11 operating system, CUDA version 11.8, Python 4.0 programming language, and PyTorch 2.1.0 deep learning framework. The learning rate employs an exponential decay strategy (initial value 0.01, decay rate 0.95 per epoch), with the batch size set to 256.

### 3.1. Rail Track Detection Experiments and Analysis

For rail track detection, we selected 1549 high-quality railway images from the public RailSem19 dataset [35] to validate our proposed method. The curated test set encompasses diverse scenarios including parallel and intersecting tracks under varying illumination and weather conditions. Each track centerline was annotated with continuous points positioned at the rail region centroids, subsequently fitted using cubic polynomial regression with least squares optimization. The remaining RailSem19 images were utilized as the training/validation set for BiSeNetV2 network, with input resolution configured to 1280 × 640 pixels to enhance segmentation performance.

**Figure 11:**
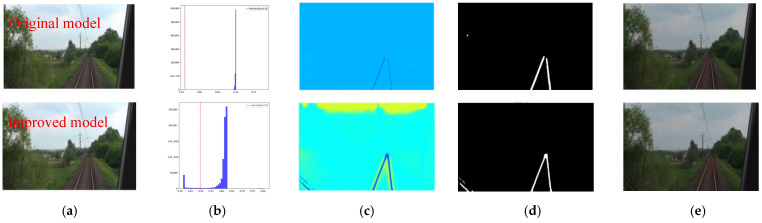
Visualized detection performance in single-pair parallel tracks configuration. (**a**) Original image. (**b**) Intensity histogram. (**c**) Heatmap. (**d**) Segmented binary mask. (**e**) Fitted curve.

To more intuitively demonstrate the detection performance of the proposed method in this paper, several typical scenarios were selected from the test set to visualize the rail line detection results, including single-pair parallel rail scenarios, multiple-pair parallel rail scenarios, and intersecting rail scenarios. The detection results before and after improvement are shown in Figure 11. In the single-pair parallel rail scenario, the pixel histogram of the rail lines exhibits a pronounced bimodal characteristic (Figure 11b), and the heatmap displays high-confidence responses in the rail regions (Figure 11c). The improved segmentation binary map clearly and completely presents two continuous rail lines with uniform widths. The fitting results are represented by two precisely parallel straight lines that closely align with the segmentation (Figure 11e), proving that the improved network demonstrates superior performance in terms of segmentation continuity and geometric relationship reconstruction for single rail lines.

**Figure 12:**
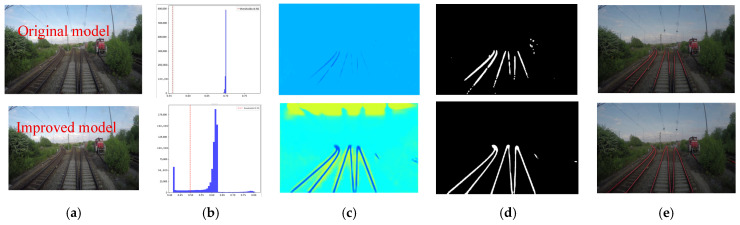
Detection results for the scenario with multiple pairs of parallel tracks. (**a**) Original image. (**b**) Intensity histogram. (**c**) Heatmap. (**d**) Segmented binary mask. (**e**) Fitted curve.

When confronted with scenarios featuring multiple pairs of parallel rails, a comparative analysis of detection results before and after improvement is presented in Figure 12. The segmentation binary map generated by the pre-improvement model exhibits fractures and discontinuities (Figure 12d), with notably poor fitting performance. In contrast, the post-improvement model’s segmentation binary map displays multiple independent, parallel, and bright white rail lines without any adhesion or breaks. The heatmap (Figure 12c) maintains high response values across each rail region. The fitting diagram (Figure 12e) successfully generates multiple parallel straight lines, each corresponding to a segmented rail, demonstrating that the proposed network model can effectively separate multiple rails, preserve their parallel relationships, and avoid cross-interference.

**Figure 13:**
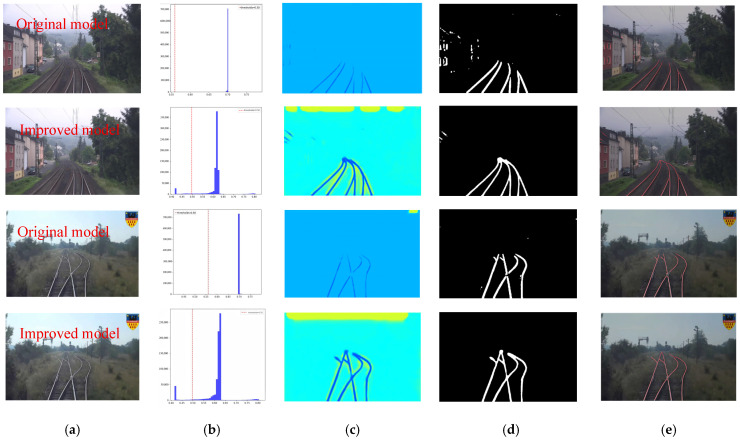
Detection results for the intersecting track scenario. (**a**) Original image. (**b**) Intensity histogram. (**c**) Heatmap. (**d**) Segmented binary mask. (**e**) Fitted curve.

For the most challenging scenario of intersecting rail tracks, a comparative analysis of detection results before and after improvement is depicted in Figure 13. Prior to improvement, the segmentation binary map (Figure 13d) exhibits line fractures or blob-like confusion at the intersecting points of the rail tracks, failing to fully represent the rail structure. The fitting diagram is unable to generate complete intersecting straight lines, thus failing to restore the “X” or “+” shaped geometric relationships. In contrast, the proposed network model clearly displays the intersecting rail tracks in the segmentation binary map, with connected lines at critical intersection points without any fractures or blob-like confusion. The heatmap (Figure 13c) still effectively characterizes the rail structure in the intersecting regions. The fitting diagram (Figure 13e) accurately generates intersecting straight lines, restoring the “X” or “+” shaped geometric relationships. This validates the network’s capability to handle complex intersecting structures, particularly in maintaining topological connectivity at intersection points.

In summary, the BiSeNetV2 network demonstrates significant advantages in railway scene segmentation tasks. Through experimental validation on the public RailSem19 dataset, in single-track scenarios, the network precisely captures subtle features along the edges of railway tracks via its detail branch. In scenarios with dense parallel tracks, the semantic branch effectively distinguishes adjacent tracks, preventing mis-segmentation caused by excessively small gaps between tracks. In track intersection scenarios, the dual-stream fusion mechanism successfully maintains the topological continuity of the tracks, with intersection point localization errors kept below 2 pixels. The aforementioned experimental results quantitatively verify the effectiveness and robustness of the improved BiSeNetV2 in real-world railway scenes, providing a reliable foundation for its deployment in real-time systems such as track condition monitoring and foreign object intrusion detection.

### 3.2. Foreign Object Detection Experiments and Analysis

#### 3.2.1. Description of Dataset and Evaluation Protocol

**Figure 14:**
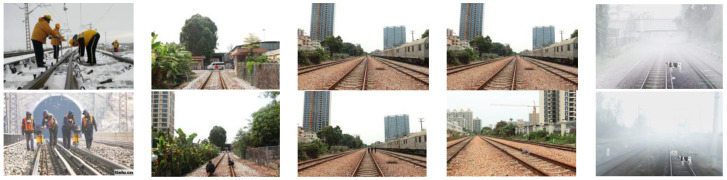
Sample images of four categories of foreign object intrusions (partial).

Since there is currently no publicly available dataset for railway track foreign object intrusion, drawing on the approach in reference [36], the sample library was established through a combination of on-site railway collection and simulated intrusion collection. Given the scarcity of real intrusion cases, simulated intrusion served as the primary method. Typical intrusion scenarios were simulated at the Guangzhou EMU Depot test site and on actual railway lines. Scenes of animal intrusion were synthesized via computer techniques, resulting in the creation of four categories of foreign object intrusion sample images, as illustrated in Figure 14.

The annotation was carried out using the open-source tool labelImg 1.5.1, with sample categories encompassing vehicles, pedestrians, animals, and falling rocks, totaling 5370 images. The constructed dataset is presented in Table 5. The foreign object detection dataset adheres to the VOC format and is divided into training, validation, and test sets in a ratio of 7:2:1.

For railway track intrusion detection tasks, where images contain two types of labels (background and objects) and prediction boxes are classified as either correct or incorrect, the evaluation process generates four sample categories: $T_{p}$ (True Positives)—correctly identified intrusions, $F_{p}$ (False Positives)—background areas mistakenly detected as objects, $T_{N}$ (True Negatives)—correctly ignored background regions, and $F_{N}$ (False Negatives)—missed intrusion objects. Based on this classification framework, the following evaluation metrics were employed [37]:

1. The mean Average Precision (mAP) serves as the primary metric for evaluating model performance in this study, representing the mean value of Average Precision (AP) scores across all detection categories.

(18) $$mAP=\frac{\sum_{n=1}^{N}A_{p}(n)}{N}$$

2. Recall serves as the metric for evaluating the model’s capability to correctly detect target objects.

(19) Recall=TPTP+FN

3. Precision measures the accuracy of the model’s predictions.

(20) $$\mathrm{Pr}ecision=\frac{TP}{TP+FP}$$

4. The detection speed was quantitatively compared using the frames-per-second (FPS) metric.

(21) $$FPS=F/t_{e}$$

In the evaluation framework: $T_{p}$ represents true positive cases where actual intrusions are correctly detected, $F_{p}$ denotes false positive scenarios where the system erroneously detects intrusions in normal conditions, $T_{N}$ indicates true negative cases where the absence of intrusions is properly identified, $F_{N}$ signifies false negative instances where existing intrusions are missed, and $t_{e}$ measures the total processing time required for detection.

#### 3.2.2. Experimental Analysis of Foreign Object Detection

(1) Experimental Analysis

An experiment was conducted on the dilation rates (r = 1, 2, 3) of DilateFormer using our self-built dataset, with the results summarized in Table 6. When r = 1, the dilated convolution configuration of DilateFormer degenerates into a standard convolution operation. Firstly, the dense sampling mechanism of r = 1 fully preserves the local correlation of input features, avoiding feature distortion caused by interval sampling when r > 1. For instance, when r = 2, only 5 non-zero points are sampled, and such sparse sampling can disrupt the fine-grained spatial information required for small object detection. Secondly, experimental data (Table 6) indicates that the r = 1 configuration achieves an mAP of 84.16% on the self-built dataset, which is 0.3% and 0.7% higher than r = 2 and r = 3 configurations, respectively, while the inference speed only decreases by 5.1%. This demonstrates that it achieves an optimal balance between accuracy and speed. Finally, the equivalent receptive field of r = 1 is 3 × 3, which forms a natural transition with the subsequent 5 × 5 and 7 × 7 convolutions in the hierarchical structure. This avoids information redundancy caused by the sudden expansion of the receptive field when r > 1. This hierarchical design enables the model to achieve multi-scale feature fusion at a lower computational cost. Therefore, r = 1 brings the most effective network performance improvement to DilateFormer, achieving a balance between accuracy and efficiency.

To validate the effectiveness of the improved ShuffleNetV2 backbone network, comparative experiments were conducted against both the baseline DarkNet-53 and the original ShuffleNetV2 on the dataset, with results presented in Table 7. Replacing DarkNet-53 with the lightweight ShuffleNetV2 reduced model parameters by 5.3 × 10^6^ and computational costs by 8.4 × 10^9^ operations while increasing frame rate by 88 FPS, albeit with a marginal 0.5% mAP decrease. These results demonstrate ShuffleNetV2’s superior lightweight characteristics but reveal its inherent challenge in balancing detection accuracy and model efficiency. Addressing this limitation, our enhanced ShuffleNetV2 incorporates SENet modules, achieving a 1.2% mAP improvement over the baseline model with negligible increases in parameter count and computation, while maintaining equivalent inference speed to the original ShuffleNetV2. This optimized architecture therefore demonstrates both theoretical rationality and practical superiority in simultaneously optimizing accuracy, efficiency, and lightweight design.

(2) Ablation Study

Using YOLOv8s as the baseline model, we conducted four ablation experiments on our custom dataset to validate the effectiveness of each proposed module. YOLOv8s-S denotes replacing the original backbone with our improved ShuffleNetV2 network, while YOLOv8s-T indicates integrating the DilateFormer module at the backbone’s terminal stage. As shown in Table 8, replacing the backbone with improved ShuffleNetV2 (YOLOv8s-S) yields a 1.2% mAP improvement on our dataset while doubling the inference speed to 215 FPS, with model parameters and FLOPs reduced to only 59.8% and 75.8% of the baseline, respectively. The added DilateFormer module (YOLOv8s-T) employs multi-scale dilated convolutions to expand the receptive field without sacrificing feature resolution, enhancing contextual perception while introducing negligible computational overhead. Our final configuration, ST-YOLOv8s, combines both modules to achieve a 3.3% mAP gain and 136 FPS improvement over the baseline, with only 72.7% parameters and 72.1% FLOPs of YOLOv8s. These systematic ablations confirm our method’s ability to optimally balance accuracy, speed, and lightweight design.

(3) Comparative Experiment Analysis

To verify the superiority of the proposed algorithm, comparative experiments were conducted under identical conditions between the improved algorithm and other methods on our custom dataset. As shown in Table 9, the ST-YOLOv8s algorithm demonstrates significant advantages on the custom railway foreign object dataset. Compared with Faster R-CNN, SSD, YOLOv3, YOLOv3-tiny, YOLOv4, YOLOv4-tiny, YOLOv5s, YOLOv7 and the baseline model YOLOv8s, the ST-YOLOv8s algorithm achieves higher detection accuracy with mAP reaching 84.9%, while maintaining relatively low parameters and computational costs. The detection speed reaches 154 FPS, outperforming other compared algorithms. Therefore, the ST-YOLOv8s algorithm achieves better balance between detection accuracy, speed, and lightweight design, demonstrating high generalizability and practical value.

**Figure 15:**
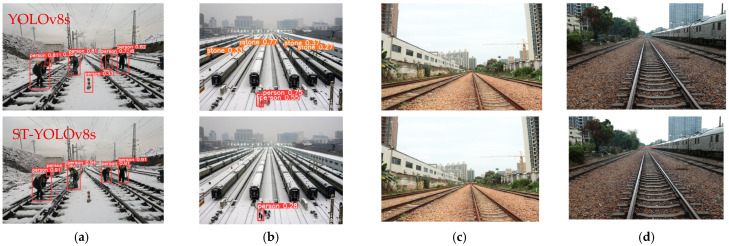
Comparison of visualization results between the ST-YOLOv8s algorithm and YOLOv8s algorithm. (**a**) False positives for near-range targets. (**b**) Missed and false detections for near-range targets. (**c**) Missed detections for far-range targets. (**d**) Missed and false detections for far-range targets.

To visually compare the detection performance between the proposed algorithm and the baseline for railway track intrusion detection, we conducted qualitative analysis using ST-YOLOv8s and YOLOv8s models on our dataset, with visualization results shown in Figure 15. For nearby intrusion targets (Figure 15a,b), our algorithm effectively reduces missed detections and false alarms compared to the baseline model. For distant targets (Figure 15c,d), the proposed method also demonstrates improved detection accuracy with fewer errors. While some missed detections remain (particularly for extremely distant targets), the ST-YOLOv8s algorithm generally detects most intrusion objects with higher confidence scores. The visualization results confirm that our proposed ST-YOLOv8s exhibits stronger shallow feature extraction capabilities, significantly reducing both missed and false detections while maintaining superior overall detection performance.

### 3.3. Quantitative Risk Assessment Experiment for Foreign Object Intrusion

**Figure 16:**
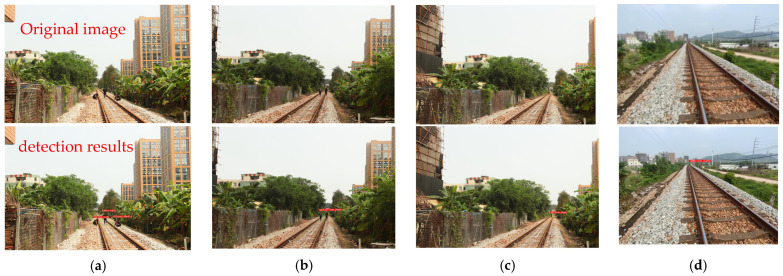
Lateral distance detection of foreign objects at different longitudinal distances. (**a**) Longitudinal distance of 20 m. (**b**) Longitudinal distance of 30 m. (**c**) Longitudinal distance of 70 m. (**d**) Longitudinal distance of 100 m.

This section focuses on quantitative risk assessment of foreign object intrusions on railway tracks, integrating lateral distance detection data with multidimensional risk evaluation metrics to establish a tiered warning system. The experiments initially collected object offset measurements at four longitudinal distances (20 m, 30 m, 70 m, and 100 m) using the lateral distance detection methodology illustrated in Figure 10, with particular emphasis on analyzing track deviation distances (in millimeters) and corresponding risk indicators for seven personnel test cases. The distances from intrusion points to both the left and right rails were calculated according to Equations (9)–(14) in the text, as demonstrated in Figure 16.

Based on the calculated lateral distances and incorporating Equations (15)–(17), the risk assessment system determines the comprehensive risk value $Z_{risk}$ of foreign object intrusion by evaluating parameters including the lateral offset risk factor $x_{risk}$ and longitudinal distance risk factor $y_{risk}$. Quantitative risk classification was performed at four longitudinal distances (20 m, 30 m, 70 m, and 100 m). Assuming personnel move laterally at 2 m/s after train whistling, analysis of the seven sample cases in Table 10 shows the following: when objects are within 100 mm of the tracks (e.g., Person2 at 150 mm from left rail), the comprehensive risk value $Z_{risk}$ reaches 0.92, triggering Level II risk; when offset exceeds 240 mm (e.g., Person2 at 241 mm), $Z_{risk}$ decreases to 0.71, downgrading to Level III. As longitudinal distance increases from 20 m to 100 m, the longitudinal risk factor $y_{risk}$ decreases from 1.49 to 0.82 (45% reduction), potentially classifying distant scenarios as low-risk. These results establish a three-tier risk prevention strategy: the Level I high-risk zone (within ±100 mm of tracks) requires immediate alarms; the Level II medium-risk zone (±100–300 mm) needs dynamic monitoring; and the Level III low-risk zone (beyond ±300 mm) allows periodic inspection. Assessment accuracy significantly decreases beyond a 70 m longitudinal distance, so we recommend high-precision sensor deployment within this range to ensure effective risk warnings.

## 4. Conclusions

Railway perimeter foreign object intrusion poses a significant threat to the operational safety of trains. Addressing the limitation of existing visual detection techniques that overly focus on object recognition while lacking risk quantification assessment, this study proposes a railway foreign object intrusion risk quantification and assessment method that integrates track semantic segmentation with spatio-temporal features, achieving a paradigm shift from object detection to risk decision-making. The main conclusions are as follows:

(1) This study achieves high-precision track modeling through an improved BiSeNetV2 network, enabling the detection of single-track, multi-track parallel, and intersecting track lines on the RailSem19 dataset. Furthermore, a lightweight ST-YOLO foreign object detection model is designed, achieving an mAP of 84.9% on a self-built dataset. Compared to the baseline, the model reduces the number of parameters and computational complexity by 27% and 28%, respectively, while increasing the FPS by 30% to 154 frames, effectively addressing the issue of missed detections of small-scale foreign objects.

(2) This paper integrates target category, lateral position risk coefficient, longitudinal distance segmented attenuation coefficient, and target dynamic velocity compensation factor into the risk assessment, establishing a comprehensive risk assessment formula. Experiments demonstrate that the model achieves quantitative risk classification for foreign object intrusion at four longitudinal distances (20 m, 30 m, 70 m, and 100 m). The model can sensitively respond to changes in foreign object positions and the influence of longitudinal distance, outputting five risk levels. This provides a quantitative decision-making basis for active train protection, significantly enhancing the intelligence level of safety protection.

(3) In this paper, when the longitudinal distance exceeds 70 m, the $y_{risk}$ coefficient decays rapidly, leading to a decrease in the sensitivity of risk assessment, primarily limited by the inherent errors of monocular vision ranging. In high-speed scenarios, i.e., when the train speed exceeds 200 km/h, image motion blur causes a decline in the foreign object detection rate. Since the existing framework does not integrate a deblurring module, future research will combine train operational status such as speed and position to more accurately achieve risk assessment for railway foreign object intrusion.

## Figures and Tables

**Table 1:** Backbone architecture of BiSeNetV2.

	Detail Branch					Semantic Branch						Output Dimensions
Input												512 × 1024
	layer	*k*	*c*	*s*	*r*	layer	*k*	*c*	*e*	*S*	*r*	
S1	Conv2d	3	64	2	1	Stem	3	16	-	4	1	256 × 512 256 × 512
	Conv2d	3	64	1	1							
S2	Conv2d	3	64	2	1							128 × 256 128 × 256
	Conv2d	3	64	1	2							
S3	Conv2d	3	128	2	1	GE	3	32	6	2	1	64 × 128 64 × 128
	Conv2d	3	128	1	2	GE	3	32	6	1	1	
S4						GE	3	64	6	2	1	32 × 64 32 × 64
						GE	3	64	6	1	1	
S5						GE	3	128	6	2	1	16 × 32 16 × 32
						GE	3	128	6	1	3	
S6						CE	3	128	-	1	1	16 × 32

**Table 2:** Risk zone classification.

Zone Type	Lateral Range (mm)	Risk Base Value
High-risk area	0 ≤ *d_x_* ≤ 717.5	1.0
Medium-risk area	717.5 ≤ *d_x_* ≤ 3157.5	0.7
Safe area	*d_x_* ≥ 3157.5	0

**Table 3:** Weights of foreign object types.

Category	Weight	Behavioral Characteristics
person	1.1	potential for sudden movement
car	1.2	bulky mass with high momentum
animal	0.9	erratic movement
stone	0.7	motionless

**Table 4:** Decision matrix for risk classification.

Risk Threshold Range	Risk Grade	Mitigation Measure
≥1.2	Level I—Extremely High Risk	Emergency Braking Application (EBA)
0.8~1.2	Level II—High Risk	Automatic Speed Constraint (ASC)
0.5~0.8	Level III—Elevated Risk	Multi-modal Warning and Brake Pre-activation (MWBP)
0.2~0.5	Level IV—Managed Risk	HUD Warning Alert
<0.2	Level V—Controlled Risk	Non-intrusive Event Logging (NIEL)

**Table 5:** Sample distribution of the dataset.

Sub-Dataset Names	Car	Person	Stone	Animal	Total
Daylight Condition Dataset	510	1270	390	612	2782
Low-Light Condition Dataset	310	750	260	170	1490
Extreme Weather Application Dataset	260	560	150	128	1098

**Table 6:** Ablation study on dilation rate configuration in DilateFormer.

r	mAP/%	Params/10^6^	FLOPs/10^9^	FPS
Baseline Model	81.6	13.2	31.5	118
r = 1	84.2	14.7	29.8	112
r = 2	83.9	14.7	29.8	107
r = 3	83.5	14.7	29.8	104

**Table 7:** Comparative experiments on improved ShuffleNetV2.

Model	mAP/%	Params/10^6^	FLOPs/10^9^	FPS
DarkNet-53	81.6	13.2	31.5	118
ShuffleNetV2	81.1	7.9	23.1	206
Proposed Method	82.8	7.9	23.9	215

**Table 8:** Ablation study results.

Model	mAP/%	Params/10^6^	FLOPs/10^9^	FPS	P/%	R/%
YOLOv8s	81.6	13.2	31.5	118	81.5	79.8
YOLOv8s-S	82.8	7.9	23.9	215	82.6	79.2
YOLOv8s-T	84.2	14.7	29.8	112	80.2	85.1
ST-YOLOv8s	84.9	9.6	22.7	154	82.1	81.2

**Table 9:** Comparative experimental analysis.

Model	mAP/%	Params/10^6^	FLOPs/10^9^	FPS
Faster R-CNN	70.8	65.2	96.1	29
SSD	68.1	49.3	85.6	41
YOLOv3	76.3	39.5	88.9	36
YOLOv3-tiny	53.6	8.4	19.5	148
YOLOv4	58.5	31.6	71.7	52
YOLOv4-tiny	59.7	7.8	24.7	139
YOLOv5s	79.4	12.2	29.2	125
YOLOv7	80.7	13.6	32.9	112
YOLOv8s	81.6	13.2	31.5	118
YOLOv12s	81.9	13.7	31.9	119
YOLOv13s	82.1	14.9	32.6	122
ST-YOLOv8s	84.9	9.6	22.7	154

**Table 10:** Quantitative risk assessment of foreign objects at different longitudinal distances.

	Distance to Left (Right) Rail		$x_{risk}$	$y_{risk}$	$Z_{risk}$	Risk Grade
Figure 16a	Person1	120.7 mm from the left rail	0.48	1.49	0.95	Level II—High Risk
	Person2	8.8mm from the left rail	0.71	1.49	1.39	Level I—Extremely High Risk
	Person3	153mm from the right rail	0.47	1.49	0.92	Level II—High Risk
Figure 16b	Person1	189mm from the right rail	0.78	0.94	0.98	Level II—High Risk
	Person2	241mm from the right rail	0.45	0.94	0.71	Level III—Elevated Risk
Figure 16c	Person1	332mm from the left rail	0.84	0.87	0.97	Level II—High Risk
Figure 16d	Person1	1280mm from the left rail	0.76	0.82	0.83	Level II—High Risk

## Data Availability

Data are contained within the article.
